# Contribution of DNA repair xeroderma pigmentosum group D genotypes to pancreatic cancer risk in the Chinese Han population

**DOI:** 10.1590/1678-4685-GMB-2017-0033

**Published:** 2017-12-18

**Authors:** Dong Yan, Xiao-Hui Liang, Wei Ding, Xin-Jian Xu, Xi-Yan Wang

**Affiliations:** 1 Department of Hepatopancreatobiliary Surgery, Affiliated Tumor Hospital of Xinjiang Medical University, Urumqi, Xinjiang, China.; 2 Department of Hypertension, First Affiliated Hospital of Xinjiang Medical University, Urumqi, Xinjiang, China.; 3 Department of Pancreatic Surgery, First Affiliated Hospital of Xinjiang Medical University, Urumqi, Xinjiang, China.; 4 Department of Xinjiang Research Institute of Cancer Prevention and Control, Affiliated Tumor Hospital of Xinjiang Medical University, Urumqi, Xinjiang, China.

**Keywords:** Pancreatic neoplasm, human xeroderma pigmentosum group D, polymorphism, smoking

## Abstract

This study aimed to determine the association between the polymorphisms and haplotypes in the xeroderma pigmentosum group D (*XPD*) gene and the risk of pancreatic cancer in the Chinese Han population. SNaPshot was used for genotyping six SNP sites of the *XPD* gene. Comparisons of the correlations between different genotypes in combination with smoking and the susceptibility to pancreatic cancer were performed. Individual pancreatic cancer risk in patients who carry mutant C alleles (AC, CC, and AC+CC) at rs13181 increased (*p* < 0.05). Taking non-smoking individuals who carry the AA genotype as a reference, and non-smoking individuals who carry mutant allele C (AC+CC), the risk of pancreatic cancer increased by 3.343 times in individuals who smoked ≥ 20 cigarettes daily, 3.309 times in individuals who smoked ≥ 14 packs per year, 5.011 times in individuals who smoked ≥ 24 packs per year, and 4.013 times in the individuals who smoked ≥ 37 packs per year (*P* < 0.05). In addition, haplotype analysis revealed that haplotype AGG, which comprised rs13181, rs3916874 and rs238415, was associated with a 1.401-fold increase in pancreatic cancer risk (*p* < 0.05). We conclude that the polymorphism of XPD Lys751Gln (rs13181) in combination with smoking contributes to increased risk of pancreatic cancer in the Chinese Han population. Haplotype AGG might be a susceptibility haplotype for pancreatic cancer.

## Introduction

Pancreatic cancer (PC) is highly malignant, has an insidious onset, and lacks early diagnostic methods. Furthermore, more than 80% of patients have lost their chance of surgery when they first visit a doctor, and overall five-year survival rate is approximately 5% (Siegel *et al.*, 2013). To date, the exact mechanism of PC remains unknown. Smoking, type 2 diabetes mellitus (T2DM), body mass index, alcohol consumption, and family history are the most consistent epidemiological risk factors for PC (Larsson *et al.*, 2007; Maisonneuve and Lowenfels,2010; Jacobs *et al.*, 2010). Multiple cohort and case-control studies have consistently demonstrated an association between PC and cigarette smoking, particularly among heavy smokers (Iodice *et al.*, 2008; Bosetti *et al.*, 2012).

A study reported that carcinogens from cigarettes may reach the pancreas via blood, or may return via bile, so contents of DNA-carcinogen adducts in smokers are higher than in non-smokers (Talamini *et al.*, 2010). The human xeroderma pigmentosum group D (*XPD*) gene can repair damage induced by bulky DNA adducts and maintain genomic stability. XPD are the major components of nucleotide excision repair (NER) pathway and transcription factor IIH (TFIIH), which are involved in gene transcription and NER by unwinding DNA around the lesion (Benhamou and Sarasin, 2005). It has been reported that mutations of the XPD gene diminish its helicase activity, resulting in defective NER capacity for bulky DNA adducts and transcriptional activity, and in an abnormal response to apoptosis (Taylor *et al.*, 1997). Mutations and defects in *XPD* gene may be closely related to tumorigenesis. The most widely investigated *XPD* polymorphism in association with cancer susceptibility comprise a non-synonymous A to C substitution in exon 23 causing a lysine (Lys) to glutamine (Gln) substitution in codon 751 (Lys751Gln, rs13181) (Shen *et al.*, 1998; Benhamou and Sarasin, 2002). Some studies have reported significant associations between codon 751 variants and predisposition to many types of cancer, including melanoma (Kertat *et al.*, 2008), lung (Wu and Ding, 2014), head and neck (Yuan *et al.*, 2011), bladder (Xiong *et al.*, 2014), and breast (Yan *et al.*, 2014). The variant genotypes are both associated with lower DNA repair capacity and a higher level of DNA adducts left in the genome (Shen *et al.*, 1998; Lunn *et al.*, 2000; Benhamou and Sarasin, 2002). A few studies investigated the association of *XPD* genotype with PC risk (Jiao *et al.*, 2007; McWilliams *et al.*, 2008). However, the results of these reports remain inconclusive and none investigated the Chinese Han population, which is genetically conservative and different from Western populations.

Tagging SNP (tag-SNP) can improve validity of analyzing the correlation between candidate genes and disease. In the current study, to better understand the pivotal roles of XPD, we adopted tag-SNP with SNP to enroll functional site - codon 751. Because of the known ethnic variation in PC risk and genotype distribution, the current study focused on the Chinese Han population only, and analyzed the contribution of *XPD* genotypes to PC susceptibility and their interactions with smoking and other clinical factors.

## Materials and Methods

### Study subjects

A total of 226 patients with pancreatic cancer, who were admitted in the Affiliated Tumor Hospital of Xinjiang Medical University and the First Affiliated Hospital of Xinjiang Medical University from December 2007 to August 2015, were enrolled in this study. Among these patients, 140 underwent pancreaticoduodenectomy, 23 underwent ^125^I implantation and palliative surgery (biopsy was performed during surgery), 59 underwent pancreatic body and tail (combined with spleen) resection, and four underwent CT-guided fine needle puncture biopsy. All patients were confirmed as having PC by histopathological examination. Two hundred and sixty-three subjects, who were admitted in the First Affiliated Hospital of Xinjiang Medical University during the same period and had no previous history of pancreatic disease were assigned as controls after initial random sampling. The exclusion criteria of the case and control groups included previous malignancy, metastasized cancer from other or unknown origin, and incomplete general information.

All enrolled participants were volunteers from the Chinese Han population. They completed a questionnaire and provided peripheral blood samples. Demographic information including age, gender, smoking and drinking status and other factors were obtained through a structured questionnaire interview. Subjects with continuous or cumulative smoking history for six months or more were defined as smokers, and the cumulative amount of smoking was calculated as packs/year = daily smoking amounts/20 x years of smoking. Drinking was defined as having at least one drink every week for more than six months (Qian *et al.*, 2014). Body mass index (BMI) was calculated from height and weight using the BMI formula (BMI=weight in kilogram divided by the square of height in meters). Diabetes and family medical history were obtained by self-report using a questionnaire at the time of enrollment. All patients signed an informed consent. The study protocol was approved by the Ethical Committee of the First Affiliated Hospital of Xinjiang Medical University.

### Blood collection and DNA extraction

Three milliliters of peripheral blood collected from each participant was placed in EDTA tubes, and stored in a freezer at −80 °C within 30 min after it was collected. The genomic DNAs were extracted from blood samples using a DNA blood kit (BioTeke, Beijing, China), according to manufacturer's instructions.

### SNP selection and genotyping

According to the Hapmap database (http://hapmap.ncbi.nlm.nih.gov/), data were analyzed using HaploView 4.0 software, minor allele frequency was adjusted to 0.15, the lower limit of linkage disequilibrium (D’) was greater than 0.8, and an r^2^ of 0.8 was selected as the threshold for the analyses. As a result, 5 tag-SNPs (rs3916874, rs238415, rs50872, rs50871 and rs238406) were selected from the HapMap covering the *XPD* gene. XPD codon 751 (rs13181), which is the most studied in the literature, was the functional site that encoded non-synonymous amino acid changes, and was enrolled into this study as well.

PCR primers and extended primers (Table 1) were designed using the Primer5 software. PCR assays were set up as follows: 1 μL of 10 x hot start *Taq* buffer, 1.2 μL of MgCl_2_, 1.5 μL of deoxy-ribonucleoside triphosphate (dNTP) mixture, 0.2 μL of hot start *Taq* DNA polymerase (Qiagen, GER), 1 μL of sample DNA, 1 μL of multiple PCR primers, and ultra pure water was added to produce 10 μL. Reaction conditions were as follows: pre-denaturation at 95 °C for 2 min, 11 cycles of 94 °C for 20 s, 65 °C for 40 s and 72 °C for 90 s, followed by an extension step at 72 °C for 2 min and cooling to 4 °C. PCR products were purified using an Exo/SAP protocol (Applied Biosystems, Foster City, CA, USA) according to the manufaccturers instructions. The PCR products were labeled by the alkaline phosphatase-labeled streptavidin biotin/exonuclease I (*Exo*I) method, using 1 U of shrimp alkaline phosphatase (SAP; Promega, Madison, WI, USA) and 1 U of *Exo*I (New England Biolabs, Beverly, MA, USA). The labeling reaction was performed at 37 °C for 1 h, followed by inactivation at 75 °C for 15 min. The SNaPshot extension reaction system consisted of 5 μL of the SNaPshot multiple reaction kit agents (Applied Biosystems), 2 μL of purified multiple PCR products, and 1 μL of the extension primer mixture, and completing the volume to 10 μL with ultra pure water. The PCR cycling conditions of the primer extension reaction were as follows: pre-denaturation at 96 °C for 1 min, followed by 28 cycles of 96 °C for 10 s, 50 °C for 5 s and 60 °C for 30 s, followed by an extension step at 60 °C for 1 min, and cooling to 4 °C. Next, 1 U of SAP was added to the 10 μL extension product and the mixture was incubated at 37 °C for 1 h then inactivated at 75 °C for 15 min. For data analysis, 0.5 μL of the purified extension product were mixed with 0.5 μL of fluorescent internal standard and 9 μL of formamide reagent, and denatured at 95 °C for 5 min. The products were sequenced using an ABI 3130XL automatic DNA sequencer (Applied Biosystems) and analyzed using the GeneMapper 4 software.

**Table 1:** PCR primers of *XPD* gene and sequences of single nucleotide primer extension.

Locus	PCR primer of *XPD* gene 5’-3’	Sequences of single nucleotide primer extension 5’-3’
rs13181	F:AACCAGGGCCAGGCAAGACT	SF:TTTTTTTTTTTTTTTTTTTTTTTTTTTTTT TTTCTGAGCAATCTGCTCTATCCTCT
	R:CCCCCTCTCCCTTTCCTCTGT	
rs3916874	F:CTGCACAGGTGGCTGTGTGTTT	SF:TTTTTTTTTTTTTTTTTTTTTTTTTTTTTTTT TTTTTTTTTGGCTCCACACTGTCTCTATTGTA
	R:GATTGGTGGAGGGGCTGACTGT	
rs238415	F:GACATCGGCCTTGTGCTTCAAT	SF:TTTTTTTTTTTTTTTTTTTTTTTTTT TTTTTGTGACCCAGTCCCCACAGC
	R:CCTTGACCCCCTACTTCACACC	
rs50872	F:TTCCCATCTGCCAACACCTCAT	SF:CCTCCCCCTCATCCTTAGG
	R:GAGGGCTTCCTGGAGGACAAGA	
rs50871	F:TTCCCATCTGCCAACACCTCAT	SR:TTTTTTTTTTTTTTTTTTTTTTTTTTTTTT TTTTTTGAAGGATGATCCAGGTGAAAGA
	R:GAGGGCTTCCTGGAGGACAAGA	
rs238406	F:GGACAAGAGTGCCAGGGGTCAG	SR:TTTTTTTTTTTTTTTTTTTTTTTTTTTT TTTTTTTTCAGCCTGCCCCACTGCCG
	R:GGCGCAGTACCAGCATGACAC	

F: upstream primer, R: downstream primers, SF: forward sequence, SR: backward sequence.

### Statistical analysis

SPSS 19.0 statistical software was used for statistical analysis. The Hardy-Weinberg equilibrium was tested for all SNPs in the control group. Chi-squared tests were used to assess differences in the distribution of genotypes and the SNP alleles between the case and control groups. Odds ratios (ORs) and 95% confidence intervals (CIs) were calculated using multivariate unconditional logistic regression with adjustments for age and gender. Haploview software version 4.0 was used to generate linkage disequilibrium (LD) plots and assess the association between haplotypes and PC. A *p*-value < 0.05 was considered to be statistically significant.

## Results

### General characteristics

Demographic characteristics and related risk factors of the subjects are shown in Table 2. The *X* ^2^-test showed that differences in age, gender, alcohol, and BMI between the case group and control group were not statistically significant (*p* > 0.05); there was also no significant difference with respect to diabetes history and family history of cancer (*p* > 0.05). Therefore, the effects of confounding factors on the results were avoided.

**Table 2:** Comparison of clinical characteristics in pancreatic cancer patients and controls.

Clinical characteristics (cases)	Patients group 226	Control group 263	*χ* ^2^ value	*p*
Age (years old)			0.146	0.986
≤ 49	32	39		
50~59	40	49		
60~69	76	87		
≥ 70	78	88		
Sex			1.335	0.248
Male	138	147		
Female	88	116		
Alcohol consumption			3.120	0.077
Yes	94	89		
No	132	174		
Diabetes history			1.672	0.196
Yes	51	47		
No	175	216		
BMI(kg/m^2^)			3.198	0.362
< 18.5	64	59		
18.5~23.9	83	97		
24~27.9	47	69		
≥ 28	32	38		
Family history of cancer			1.732	0.188
Yes	31	26		
No	195	237		

BMI = body mass index P-values were calculated from two-sided Chi-squared tests.

P-values were calculated from two-sided Chi-squared tests.

### The correlation of smoking and PC

The risk of PC did not increase in smokers compared with non-smokers (OR=1.384, 95% CI=0.896-2.259, *p*=0.150), but the risk of PC increased in patients whose daily smoking amount was more than 20 cigarettes (OR=1.569, 95% CI=1.005-2.448, *p*=0.047). According to the cumulative smoking amount, the risk of PC was increased by 0.767 (OR=1.767, 95% CI=1.020-3.063, *p*=0.041) in patients who smoked ≥ 14 packs per year, by 0.856 (OR=1.856, 95% CI=1.000-3.445, *p*=0.048) in patients who smoked ≥ 24 packs per year, and by 1.209 (OR=2.209, 95% CI=1.041-4.686. *p*=0.035) in patients who smoked ≥ 37 packs per year (Table 3).

**Table 3:** Relationship between smoking and pancreatic cancer.

Smoking	Patients group (cases)	Control group (cases)	OR	95% CI		*χ* ^2^	p
				Lower limit	Upper limit		
Smoking							
No	126	167	1.00				
Yes	100	96	1.381	0.960	1.985	3.037	0.081
Amount of cigarettes a day							
0 (no smoking)	126	167	1.00				
≤ 9	15	16	1.243	0.592	2.608	0.331	0.565
10~19	27	31	1.154	0.656	2.032	0.248	0.619
≥ 20	58	49	1.569	1.005	2.448	3.960	0.047
Total (packs per year)							
0 (no smoking)	126	167	1.00				
< 14	16	37	0.573	0.305	1.077	3.046	0.081
14~23.9	36	27	1.767	1.020	3.063	4.180	0.041
24~36.9	28	20	1.856	1.000	3.445	3.914	0.048
≥ 37	20	12	2.209	1.041	4.686	4.432	0.035

CI = confidence interval; OR = odds ratio;

P values were calculated from two-sided chi-squared tests.

### Correlation analysis of PC susceptibility

The genotype distributions of six SNP loci in the case and control groups were all in accordance with the Hardy-Weinberg equilibrium (*p* > 0.05, Table 4). The C allele frequency of the rs13181 site in patients with PC in the case group was higher than in the control group (*p*=0.005). Compared with individuals carrying the AA genotype, the risk of PC increased in patients carrying the mutant C allele (AC, CC, and AC+CC) was *p*=0.044, *p*=0.040, and *p*=0.012, respectively. There was no significant difference in the genotype and allele frequency distributions of the five selected tag-SNP loci between the case group and control group (*p* < 0.05; Tables 4 and 5).

**Table 4:** Characteristics of the 6 SNPs in *XPD* Gene.

SNP ID	Chromosome position	location	Base change	MAF		HWE *p*		*p*
				case	control	case	control	
rs13181	45854919	Extron23	A/C	0.32301	0.24144	0.521	0.745	0.005
rs3916874	45856926	Intron17	G/C	0.13496	0.16160	0.662	0.355	0.244
rs238415	45857235	Intron17	C/G	0.35840	0.34791	0.189	0.113	0.732
rs50872	45862449	Intron12	C/T	0.15708	0.19962	0.999	0.607	0.084
rs50871	45862515	Intron12	T/G	0.29203	0.29658	0.974	0.588	0.877
rs238406	45868309	Extron6	C/A	0.32079	0.32889	0.162	0.251	0.787

MAF = Minor Allele Frequency, HWE = Hardy-Weinberg Equilibrium.

P values were calculated from two-sided chi-squared tests.

**Table 5:** Association between polymorphisms of *XPD* gene in pancreatic cancer patients from the Chinese Han population.

SNP	Genotype	Case	Control	OR (95%CI)	*p*	OR (95%CI) †	*p* †
rs13181	AA	118	167	1.00(reference)			
	AC	70	65	1.524(1.010~2.301)	0.044	1.535(1.015-2.320)	0.042
	CC	38	31	1.735(1.021~2.946)	0.040	1.707(1.003-2.903)	0.049
	AC+CC	108	96	1.592(1.108~2.287)	0.012	1.596(1.109-2.298)	0.012
rs3916874	GG	167	181	1.00(reference)			
	GC	57	79	0.782(0.524-1.167)	0.228	0.778(0.520-1.165)	0.223
	CC	2	3	0.723(0.119-4.378)	0.723	0.782(0.127-4.826)	0.791
	GC+CC	59	82	0.780(0.525-1.158)	0.217	0.781(0.524-1.163)	0.223
rs238415	CC	102	123	1.00(reference)			
	CG	86	97	1.069(0.723-1.581)	0.738	1.504(1.017-2.224)	0.056
	GG	38	43	1.066(0.640-1.773)	0.807	1.303(0.772-2.198)	0.322
	CG+GG	124	140	1.068(0.748-1.526)	0.718	1.444(1.004-2.076)	0.051
rs50872	CC	161	172	1.00(reference)			
	CT	59	77	0.819(0.548-1.223)	0.328	0.823(0.550-1.232)	0.344
	TT	6	14	0.458(0.172-1.220)	0.110	0.677(0.414-1.105)	0.118
	CT+TT	65	91	0.763(0.520-1.120)	0.167	0.766(0.521-1.126)	0.175
rs50871	TT	112	135	1.00(reference)			
	TG	96	100	1.157(0.795-1.685)	0.446	1.139(0.781-1.661)	0.500
	GG	18	28	0.775(0.407-1.474)	0.436	0.904(0.653-1.251)	0.542
	TG+GG	114	128	1.074(0.752-1.532)	0.696	1.065(0.745-1.522)	0.731
rs238406	CC	113	128	1.00(reference)			
	CA	81	97	0.946(0.641-1.395)	0.779	0.934(0.630-1.384)	0.733
	AA	32	38	0.954(0.559-1.627)	0.812	0.979(0.568-1.689)	0.940
	CA+AA	113	135	0.948(0.664-1.353)	0.769	0.945(0.660-1.354)	0.759

† P-value was calculated by unconditional logistic regression analysis adjusted for age and sex.

**Figure 1:**
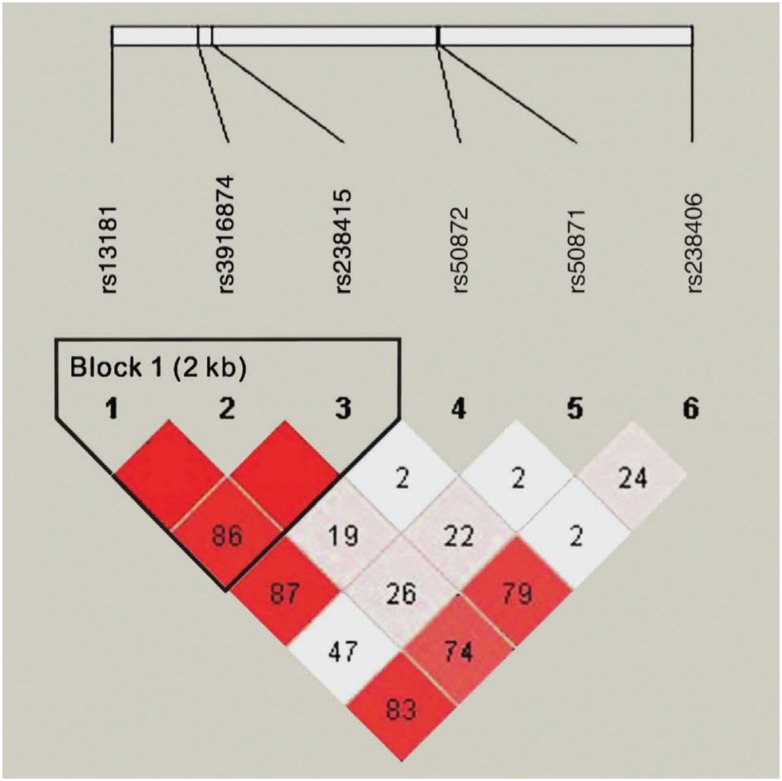
Linkage disequilibrium (LD) plot of the SNPs in *XPD*. Six SNP sites of XPD gene are shown in the upper part of the figure; the number in the box in the lower part shown the 100xD'value (D: linkage disequilibrium parameter). A standard color scheme is used to display LD with bright red for very strong LD (LOD ≥ 2, D'=1), white for no LD (LOD < 2, D' < 1), pink red for intermediate LD.

A haplotype analysis was performed to evaluate the frequencies of haplotypes based on the three polymorphisms within block 1 of *XPD*. Block 1 includes the completely linked rs13181, rs3916874 and rs238415 (Figure 1). The haplotype AGG was significantly associated with increased risk of PC (OR=1.401, 95% CI=1.065-1.844, *p*=0.016; Table 6).

**Table 6:** XPD haplotype frequencies and associations with pancreatic cancer risk in the Chinese Han population.

Haplotype	Case (%)	Control (%)	OR (95%CI)	*p*
AGG	0.479	0.402	1.401(1.065~1.844)	0.016
AGC	0.261	0.304	0.824(0.610~1.114)	0.209
ACC	0.140	0.165	0.838(0.574~1.222)	0.357
CGC	0.103	0.125	0.816(0.532~1.251)	0.350

Haplotype constructed with the order of SNPs: rs13181, rs3916874 and rs238415.

### Influence of rs13181 polymorphism combined with smoking on the risk of PC

With non-smoking individuals carrying the wild AA genotype as reference, the risk of PC did not increase in individuals carrying the AA genotype and with a smoking history (*p* > 0.05). The risk of PC increased by a factor of 0.384 in non-smokers carrying the mutant C allele and that were (AC+CC) (*p*=0.150), and in smokers carrying the mutant C allele and that were (AC+CC). Meanwhile, the risk of PC increased by 2.343 times (*p*=0.002) in individuals who smoked ≥ 20 cigarettes daily, 2.309 times (*p*=0.015) in individuals who smoked ≥ 14 packs per year, 3.013 times (*p*=0.032) in the individuals who smoked ≥ 24 packs per year, and 4.011 times (*p*=0.010) in the individuals who smoked ≥ 37 packs per year (Tables 7 and 8).

**Table 7:** Correlation between daily cigarette amounts a with rs13181-locus polymorphic genotypes and susceptibility of pancreatic cancer.

Genotype	Cigarettes a day	Patients group (cases)	Control group (cases)	OR (95%CI)†	*p* †
AA	0 (no smoking)	64	99	1.000	
	≤ 9	6	11	0.855(0.305~2.413)	0.738
	10~19	14	19	1.126(0.539~2.463)	0.729
	≥ 20	34	38	1.371(0.783~2.418)	0.266
AC + CC	0 (no smoking)	62	68	1.384(0.896~2.259)	0.150
	≤ 9	9	5	2.772(0.904~8.695)	0.071
	10~19	13	12	1.656(0.732~3.923)	0.237
	≥ 20	24	11	3.343(1.567~7.472)	0.002

† P-value was calculated by unconditional logistic regression analysis adjusted for age and sex.

**Table 8:** Relationship between total cigarettes per year with rs13181-locus polymorphic genotypes and susceptibility of pancreatic cancer.

Genotype	Total cigarettes (packs per year)	Patients group (cases)	Control group (cases)	OR (95%CI)†	*p* †
AA	0 (no smoking)	64	99	1.000	
	<14	6	23	0.392(0.147~1.062)	0.061
	14~23.9	21	20	1.620(0.809~3.178)	0.171
	24~36.9	15	16	1.424(0.665~3.122)	0.352
	≥ 37	12	9	2.071(0.867~5.181)	0.124
AC + CC	0 (no smoking)	62	68	1.431(0.987~2.256)	0.152
	<14	10	14	1.116(0.506~2.729)	0.857
	14~23.9	15	7	3.309(1.286~8.547)	0.015
	24~36.9	13	4	5.011(1.549~16.181)	0.010
	≥ 37	8	3	4.013(1.007~15.974)	0.032

† P-value was calculated by unconditional logistic regression analysis adjusted for age and sex.

## Discussion

Studies have shown that the risk of breast cancer, esophageal cancer, liver cancer, lymphoma, lung cancer and melanoma was increased in individuals carrying the XPD 751Gln variant allele (Kertat *et al.*, 2008; Yan *et al.*, 2014; Wu *et al.*, 2014; Wang *et al.*, 2015). However, the conclusions were not consistent. Studies conducted by Sun *et al.* (2015) revealed that the risk of melanoma was not increased in Caucasian populations carrying XPD 751Gln variant alleles. Furthermore, the polymorphisms of XPD Lys751Gln and Asp312Asn had no relationship with the susceptibility of non-Hodgkin's lymphoma (Chen *et al.*, 2015), and meta-analysis results revealed that the polymorphism of XPD Lys751Gln was not associated with the risk of liver cancer (Zhang and Mou, 2013).

In the present study, we investigated the association of XPD codon 751 genotypes with PC susceptibility in the Chinese Han population. The results showed that the polymorphism at 5 tag-SNP loci was not related with the genetic susceptibility to PC, but that the AC and CC genotypes of XPD codon 751 were associated with a higher risk of PC (Table 5). Allelic frequency analysis results also revealed that the C allele of XPD codon 751 was associated with a higher risk of PC (Table 4). Additionally, the haplotype AGG, which consists of rs13181, rs3916874 and rs238415, was associated with an increased risk of PC (Table 6). To the best of our knowledge, this is the first epidemiological study based on molecular genetics to determine a significant association between the XPD codon 751 genotype and the susceptibility to PC in an analysis including a genetic-lifestyle interaction. In 2007 it was first reported that the XPD codon 312 polymorphisms might be a genetic risk modifier for smoking-related PC (Jiao *et al.*, 2007). Subsequently, the XPD codon 312 polymorphisms was also shown to be associated with PC risk (McWilliams *et al.*, 2008). Since then, few reports have focused on investigating the associations of three common XPD polymorphisms (codon 156, codon 312 and codon 751) with PC risk among different ethnicities. Our positive findings for XPD codon 751 are inconsistent with previous investigations that reported that the C allele of XPD codon 751 is not a genetic risk factor.

The results of this study revealed that the risk of PC was increased in individuals carrying the mutant allele C (AC, CC, and AC+CC) at XPD Lys751Gln compared with the wild genotype AA. Taking non-smoking individuals who carry the wild genotype AA as reference, the risk of PC was significantly increased in smokers who carry allele C (AC+CC) mutations and whose smoking amounts were ≥ 20 cigarettes daily and ≥ 14 packs per year. This indicates that smoking can increase the risk of PC, and that this risk is increased with the increase in daily cigarette smoking and packs per year. Furthermore, smokers who carry the XPD 751Gln variant allele are more likely to suffer from PC.

There was an interaction between the XPD Lys751Gln polymorphism and smoking, which was consistent with the results of the meta-analysis in lung cancer performed by Feng *et al.* (2012). The reasoning in that analysis was as follows: (1) PC is a polygenic disease with a genetic predisposition, and approximately 10% of the patients with PC have a genetic background (Pancreatic surgery group of Chinese Surgical Society, 2014); (2) smoking is a recognized risk factor for PC (Tranah *et al.*, 2011; Ryan *et al.*, 2014), and tobacco contains a variety of toxic and harmful substances that can produce free radicals, which can lead to DNA damage and cell carcinogenesis (Halliwell and Whiteman, 2004; Ozguner *et al.*, 2005; Li *et al.*, 2011); (3) studies performed by Wlodarczyk and Nowicka (2012) revealed that the DNA repair ability of individuals who carry the wild homozygote Lys/Lys combination was higher than that of individuals who are heterozygous Lys/Gln or mutant homozygous Gln/Gln. The results of this study revealed that the risk of PC was increased in individuals who carry the XPD 751Gln allele, suggesting that a codon 751 mutation may lead to a decline in DNA repair capacity and increase tumor susceptibility; (4) the XPD 751 site is located in the carboxyl terminus, and its conservation is poor. The conversion of A to C (Lys to Gln) in codon 751 may affect the interaction between its protein product and p44 (a subunit of the multi-enzyme complex TF II H), reduce helicase activity, and thereby result in defects in nucleotide excision repair. This can induce the decline in transcription activity and an abnormal response to cell apoptosis, which may increase cancer susceptibility (Egly and Coin, 2011; Wlodarczyk and Nowicka, 2012); (5) the polymorphism changes at other sites in the introns were not functional polymorphisms. They only played a role in splicing, in translation bypass, or in post-translational processing. Hence, they are unlikely to affect the protein function and might not be related to the risk of PC.

In summary, in individuals who carry the mutant gene, the risk of PC might be reduced through actions like quitting smoking, smoking control in public places, and early intervention in high-risk populations. The major limitations of our study were the relatively small sample size and the inclusion of Chinese Han participants only, which limits generalizability across other populations. In the future, the combined detection of more samples, and multiple gene loci will be required. Research on the interaction between genes and environment are also needed for establishing an effective mode of screening, treatment and prevention for PC.
